# Association of genetic and behavioral characteristics with the onset of diabetes

**DOI:** 10.1186/s12889-019-7618-z

**Published:** 2019-10-15

**Authors:** Carmen D. Ng, Jordan Weiss

**Affiliations:** 1 Hubert Department of Global Health and the Emory Global Diabetes Research Center, Emory University, Atlanta, GA USA; 2 Population Studies Center and the Leonard Davis Institute of Health Economics, University of Pennsylvania, Philadelphia, PA USA

**Keywords:** Diabetes, Longitudinal studies, Polygenic score

## Abstract

**Background:**

Prior work has established sociodemographic, lifestyle, and behavioral risk factors for diabetes but the contribution of these factors to the onset of diabetes remains unclear when accounting for genetic propensity for diabetes. We examined the contribution of a diabetes polygenic score (PGS) to the onset of diabetes in the context of modifiable known risk factors for diabetes.

**Methods:**

Our sample consisted of 15,190 respondents in the United States-based Health and Retirement Study, a longitudinal study with up to 22 years of follow-up. We performed multivariate Cox regression models stratified by race (non-Hispanic white and non-Hispanic black) with time-varying covariates.

**Results:**

We observed 4217 (27.76%) cases of incident diabetes over the survey period. The diabetes PGS was statistically significantly associated with diabetes onset for both non-Hispanic whites (hazard ratio [HR] = 1.38, 95% confidence interval [CI] = 1.30, 1.46) and non-Hispanic blacks (HR = 1.22, 95% CI = 1.06, 1.40) after adjusting for a range of known risk factors for diabetes, highlighting the critical role genetic endowment might play. Nevertheless, genetics do not downplay the role that modifiable characteristics could still play in diabetes management; even with the inclusion of the diabetes PGS, several behavioral and lifestyle characteristics remained significant for both race groups.

**Conclusions:**

The effects of genetic and lifestyle characteristics should be taken into consideration for both future studies and diabetes management.

## Background

Diabetes is the seventh leading cause of death in the United States (US) and has been implicated in the etiology of several other leading causes of death [1]. Diabetes was responsible for more than 250,000 deaths in 2015 [2] and, in 2012, imposed an economic burden of approximately $245 billion stemming from direct medical costs and loss of productivity [3]. The expected rise in diabetes prevalence among the US adult population, from 14% in 2010 to an estimated 21% in 2050 [4], will impose even greater burdens on the nation’s economic and healthcare systems, as well as patients and their families.

Age, smoking behavior, body mass index (BMI), and levels of physical activity have all been implicated as risk factors for diabetes [5]. Family history and genetic variants have also been linked to increased diabetes risk [6], but it has been suggested that their influence on diabetes is greatest for middle-aged individuals between the ages of 35 and 60 [7], plausibly suggesting an increased importance of behavioral or lifestyle characteristics in later life for diabetes onset at older ages. Thus, exploring how modifiable risk factors and genetic risk influence diabetes onset in later life may aid in our understanding of the progression of diabetes as well as the utility of targeting specific modifiable risk factors for intervention among individuals who vary in their genetic predisposition.

Two prior studies, one using the Framingham Offspring Study [8] and one using cohorts of Swedish and Finnish subjects [9], found that genetic makeup plays a modest but significant role in predicting new cases of diabetes, even after accounting for common risk factors. These studies highlight the utility of incorporating a genetic component into analyses looking at the associations between risk factors and diabetes onset. However, these studies were each limited to fewer than 20 single nucleotide polymorphisms (SNPs). Increased collection of genetic material over the past decade has led to advances in genome-wide association studies (GWASs) and the construction of polygenic scores (PGSs) which can elucidate a better understanding of the genetic risk for diabetes.

Our aim in this study was to examine the effects of genetic risk of diabetes and later-life behavioral and lifestyle characteristics associated with diabetes. We used data from the national population-based and longitudinal US Health and Retirement Study (HRS). We conducted a time-to-event analysis with time-varying covariates to better understand how genetic endowment combines with changing behavioral characteristics to shape the risk of diabetes onset for non-Hispanic whites and non-Hispanic blacks. We hypothesized that a higher genetic predisposition for diabetes would be associated with a higher risk of diabetes onset for both race groups. Furthermore, we analyzed which behavioral and lifestyle characteristics would still have statistically significant relationships with diabetes onset even after controlling for the genetic component, as those with persisting significant associations may be the most critical in terms of clinical recommendations for diabetes management.

## Methods

### Study population

The HRS is a nationally representative and longitudinal study that has biennially assessed the financial, physical, and mental well-being of community-dwelling adults at least 50 years of age and their spouses since 1992. Since the conception of the HRS, new participants have been added to the survey. The HRS is sponsored by the National Institute on Aging (NIA U01AG009740) and is conducted by the University of Michigan [10].

From 2006 to 2012, the HRS collected genetic data from a sub-sample of non-Hispanic white and non-Hispanic black respondents who consented and provided salivary deoxyribonucleic acid (DNA). Details on the sample selection and consent procedures are available elsewhere [11]. We restricted our analysis to the non-Hispanic white and non-Hispanic black respondents with available genetic information, and followed these respondents from 1992 to 2014. We linked the HRS data files compiled by RAND Corporation [12] with the HRS genetic data containing a PGS for diabetes [11]. Descriptions of the assay and calculation procedures are detailed elsewhere [11].

### Measures

#### Outcome

Incident diabetes was determined by a respondent’s affirmative response to the question: “Since we last talked to you, that is since [last interview date], has a doctor ever told you that you have diabetes or high blood sugar?” Our outcome was the age at which individuals first reported a diabetes diagnosis. Age was censored for individuals who did not report diabetes by the last wave in 2014 or who died without ever reporting diabetes.

#### Exposure

GWASs have identified a large number of genetic variants, typically SNPs, associated with a wide range of health outcomes and behaviors. However, the majority of these variants have a small effect and typically correspond to a small fraction of truly associated variants, meaning that they have limited predictive power. A PGS aggregates and weights this information into a single measure linked to a phenotype of interest [13]. Genotypes in the HRS were assessed using the llumina HumanOmni2.5 BeadChips (HumanOmni2.5-4v1, HumanOmni2.5-8v1, HumanOmni2.5-8v1.1; Illumina, Inc., San Diego, CA, USA), which assessed more than 1.9 million SNPs after applying standard quality control procedures [14].

The diabetes PGS used in this analysis was constructed by HRS researchers based on a meta-analysis of GWASs for diabetes conducted by Morris and colleagues, which considered a large number of SNPs, more than 700,000 of which overlapped with the HRS sample; ultimately ten of these were found to be significant and used to construct the diabetes PGS [15]. SNP effect sizes were estimated among samples of primarily European ancestry using a stage one (discovery) sample of 12,171 cases of diabetes and 56,862 controls and a stage two (replication) sample of 22,669 cases and 58,119 controls [15].

The GWASs in the meta-analysis used to estimate SNP weights were derived from analyses based on European ancestry groups; in other words, the SNP weights that were developed from the European GWAS were applied to the African ancestry PGS, which may affect the predictive power and interpretation of the diabetes PGS for the sample of non-Hispanic blacks [13, 16, 17]. The PGSs were standardized by the HRS for each ethnicity to a standard normal curve (mean = 0, standard deviation [SD] = 1) [11]. This PGS z-score allowed for a simple interpretation—a one SD increase in the PGS versus the change of one risk allele within a race group. In our primary analysis, PGS was included as a continuous standardized score. In other words, a higher PGS score reflected higher genetic susceptibility to diabetes. We also performed sensitivity analyses with the PGS as a dichotomous variable (z-score < 0, z-score ≥ 0) and as a categorical variable splitting the PGS into tertiles.

#### Covariates

We selected covariates based on their anticipated association with diabetes. Sociodemographic covariates included sex (male, female), race (non-Hispanic white, non-Hispanic black), foreign born (yes, no), level of education (less than high school, high school/GED, some college, college or above), and partnership status (married/partnered, not married/partnered). Measures of economic well-being included employment status (employed, unemployed, retired, disabled, not in labor force), household income (log-transformed),^1^ household wealth (log-transformed),^2^ and whether the respondent had Medicare (yes, no), Medicaid (yes, no) or another form of health insurance (yes, no). We assessed behavioral and lifestyle characteristics by including respondent’s self-report of BMI (continuous), exercise (waves 1-6: report of vigorous activity at least three times per week; waves 7-12: report of vigorous activity more than once per week), smoking status (never smoker, current smoker, former smoker), and alcohol consumption (report of consuming 3+ alcoholic drinks on days they drank). Extreme values of BMI (BMI < 10, BMI > 75), were recoded as missing values. We also included self-reported binary indicators of whether the respondent had been diagnosed between waves with high blood pressure, cardiovascular disease, and arthritis, which are important health comorbidities for diabetes [14, 18, 19]. For the purpose of this analysis, our main interest was in the behavioral and lifestyle variables, and how they were modified with the inclusion of our exposure. By adjusting for all these sociodemographic covariates, measures of economic well-being, and health comorbidities, we attained better estimates of our behavioral and lifestyle variables.

Additionally, we adjusted for birth cohort to account for the structured sampling design of the HRS which introduces new birth cohorts approximately every six years. We also included ancestry-specific principal components to account for possible confounding from population stratification and possible ancestry differences in genetic makeup that could bias estimates, as recommended in the literature [11, 16]. See Ware et al. [11] for detailed information on the construction of the ancestry-specific principal components. Their estimates are not displayed in our tables for brevity.

### Statistical analysis

Our analytic sample consisted of 15,190 respondents, of which 12,090 were non-Hispanic white and 3100 were non-Hispanic black. Over the course of the study period, this resulted in 103,059 person-years of follow-up.

Kaplan-Meir survival curves and multivariate Cox regression models [20] were used to estimate the contribution of the diabetes PGS to diabetes onset after adjusting for time-varying measures of behavioral and lifestyle characteristics. First, models were run as a function of all covariates except for the diabetes PGS and ancestry-specific principal components, both on the analytic sample and stratified by race to account for ancestral differences between non-Hispanic whites and non-Hispanic blacks [17, 21]. Most GWASs, including the one conducted by Morris and colleagues [15], are done predominantly on observations of European descent, so the predictive ability of the PGS might differ by race. These models were then run with the addition of the genetic variables as independent variables, again, both on the analytic sample and stratified by race. This second set of models demonstrated how the relationships changed with the inclusion of the genetic components. Concordance values (i.e., the proportion of pairs of cases in which the subject with higher risk had the event before the subject with lower risk) were used as goodness of fit measures [22]. Analyses of deviance, using log likelihoods, were run between corresponding models in the first and second sets [23]. Because of the nested nature of these models, these analyses were able to determine how the inclusion of the genetic component altered model fit.

In all our survival models, we included cluster-robust standard errors to account for household stratification in the HRS and to address potential within-household spillover effects [24]. We used age as the time unit in all analyses with an individual’s age at study entry as the baseline measure. All statistical analyses were performed in R version 3.5.0 [25] with the “survival” package for our primary analyses [26]. In all cases, significance was reported at the five-percent level.

## Results

Table 1 shows summary characteristics for some basic demographics and the behavioral and lifestyle characteristics of the analytic (i.e., genetic) sample at baseline. The analytic sample was 42.04% male and 79.59% of respondents were non-Hispanic white. The mean age was 56.53 years. The non-Hispanic white sample was slightly older and more male than the non-Hispanic black sample. The mean BMI of the non-Hispanic white sample was about 27 kg/m^2^, which would be classified as overweight, while the mean BMI of the non-Hispanic black sample was about 30 kg/m^2^, which is the threshold for obese. There were fewer regular exercises among non-Hispanic blacks, but fewer current smokers and heavy drinkers among non-Hispanic whites.

**Table 1:** Summary Characteristics for the Analytic Sample and Race Sub-Samples at Baseline

Characteristic	Mean or %		
	Analytic sample (*n* = 15,190)	Non-Hispanic whites (*n* = 12,090)	Non-Hispanic blacks (*n* = 3100)
Age, mean	56.53	56.90	55.09
Male, %	42.04	42.98	38.39
BMI, mean	27.82	27.25	30.04
Regular exerciser, %	32.12	33.76	25.75
Smoking status, %			
Current smoker	21.59	19.61	29.32
Former smoker	36.08	37.54	30.41
Never smoker	42.21	42.75	40.11
Heavy drinker, %	9.33	8.97	10.71

Note. Statistically significant differences between non-Hispanic white and non-Hispanic black respondents were observed for all characteristics at the *p* = 0.05 level

**Fig. 1:**
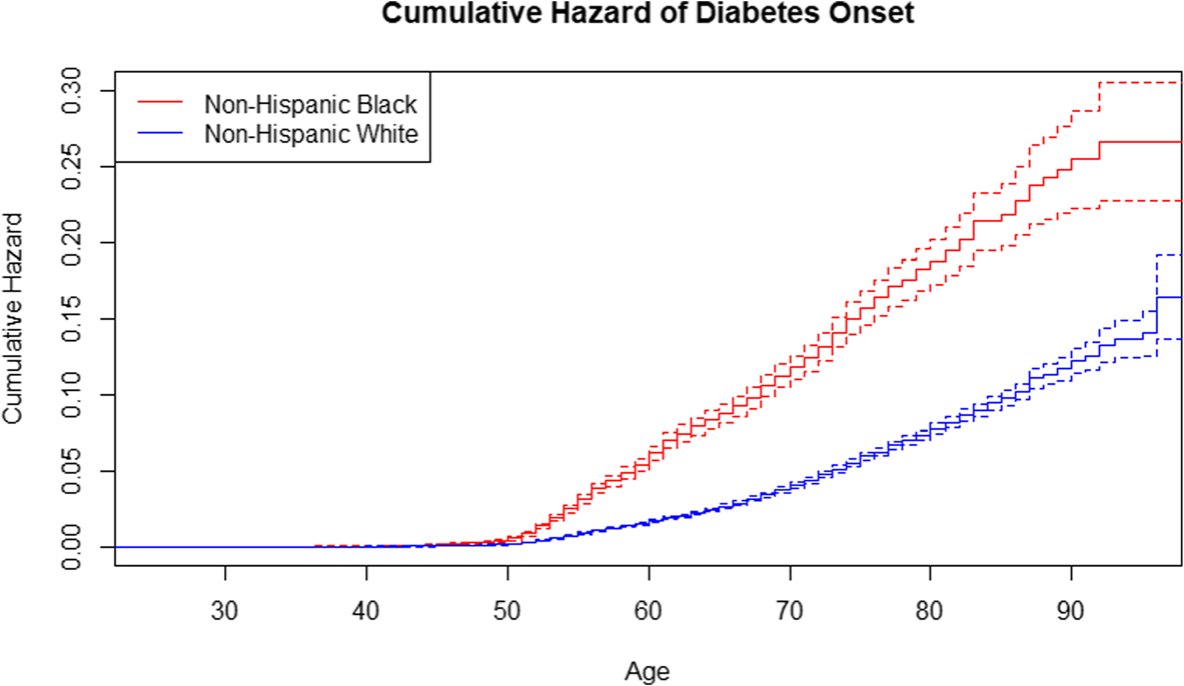
Unadjusted Cumulative Hazard of Diabetes Onset for the Analytic Sample Stratified by Race with 95% Confidence Intervals

A total of 4217 (27.76%) individuals reported being diagnosed with diabetes over the survey period. In Fig. 1, we display the unadjusted cumulative hazard of diabetes onset for non-Hispanic white and non-Hispanic black respondents. As expected, the cumulative hazard increased with advancing age. However, the curve for non-Hispanic blacks rose more quickly than that for non-Hispanic whites. By the end of the age range, the hazard of diabetes onset was clearly more likely among non-Hispanic blacks than non-Hispanic whites.

Table 2 shows the results from three separate multivariate Cox regression models for diabetes onset as a function of all covariates except the diabetes PGS and ancestry-specific principal components. The first used the analytic sample, the second used only non-Hispanic white respondents, and the third used only non-Hispanic black respondents. In the model using the analytic sample, non-Hispanic whites had a lower risk of diabetes onset relative to non-Hispanic blacks, which we demonstrated in Fig. 1. Respondents who reported being married/partnered, being disabled (compared to employed), having higher BMI, being a current smoker (compared to a never smoker), having high blood pressure, and having a cardiovascular disease were significantly associated with increased risk of diabetes onset whereas those who reported being retired (compared to employed), having higher levels of income or wealth, being a Medicare recipient, being a heavy drinker, and having arthritis were significantly associated with reduced risk of diabetes onset. Sex, foreign-born status, educational attainment, participation in Medicaid, use of other health insurance, or physical activity were not found to be significant.

**Table 2:** Hazard Ratios from Multivariate Cox Regression Models Without the Diabetes PGS Included as a Covariate

Characteristic	Hazard ratio (95% confidence interval)		
	Analytic sample (*n* = 15,190)	Non-Hispanic whites (*n* = 12,090)	Non-Hispanic blacks (*n* = 3100)
Male	1.070 (0.982, 1.166)	1.071 (0.971, 1.183)	0.981 (0.819, 1.176)
Non-Hispanic white	0.735 (0.659, 0.819)	NA	NA
Foreign-born	0.944 (0.778, 1.146)	0.892 (0.706, 1.126)	1.059 (0.749, 1.496)
Education			
Less than high school (reference group)	1	1	1
High school/GED	1.018 (0.904, 1.147)	0.986 (0.856, 1.135)	1.023 (0.821, 1.274)
Some college	1.003 (0.880, 1.143)	0.986 (0.846, 1.149)	0.980 (0.763, 1.259)
College or above	0.892 (0.773, 1.029)	0.875 (0.741, 1.033)	0.955 (0.713, 1.278)
Married/partnered	1.597 (1.447, 1.763)	1.594 (1.419, 1.790)	1.540 (1.288, 1.840)
Employment status			
Employed (reference group)	1	1	1
Unemployed	0.981 (0.772, 1.247)	0.816 (0.593, 1.123)	1.383 (0.953, 2.007)
Retired	0.599 (0.540, 0.665)	0.568 (0.503, 0.642)	0.738 (0.604, 0.902)
Disabled	1.932 (1.498, 2.491)	1.818 (1.270, 2.602)	2.247 (1.533, 3.294)
Not in labor force	0.919 (0.770, 1.097)	0.862 (0.707, 1.052)	1.189 (0.786, 1.801)
Income	0.907 (0.867, 0.950)	0.889 (0.841, 0.941)	0.953 (0.878, 1.035)
Wealth	0.920 (0.899, 0.942)	0.912 (0.885, 0.939)	0.949 (0.913, 0.987)
Medicare	0.190 (0.168, 0.214)	0.173 (0.150, 0.199)	0.253 (0.199, 0.320)
Medicaid	1.032 (0.864, 1.232)	0.993 (0.763, 1.292)	1.136 (0.893, 1.445)
Health insurance	1.034 (0.950, 1.127)	0.996 (0.903, 1.098)	1.191 (0.999, 1.420)
BMI	1.086 (1.079, 1.093)	1.098 (1.090, 1.107)	1.057 (1.045, 1.069)
Regular exerciser	1.002 (0.923, 1.089)	1.076 (0.980, 1.183)	0.833 (0.699, 0.994)
Smoking status			
Never smoker (reference group)	1	1	1
Current smoker	1.370 (1.211, 1.550)	1.382 (1.192, 1.602)	1.320 (1.044, 1.667)
Former smoker	1.076 (0.989, 1.171)	1.034 (0.940, 1.138)	1.228 (1.029, 1.465)
Heavy drinker	0.808 (0.699, 0.933)	0.779 (0.660, 0.921)	0.940 (0.705, 1.253)
High blood pressure	1.852 (1.700, 2.016)	1.898 (1.724, 2.090)	1.564 (1.290, 1.896)
Cardiovascular disease	1.112 (1.016, 1.216)	1.142 (1.032, 1.263)	1.053 (0.858, 1.294)
Arthritis	0.790 (0.729, 0.855)	0.771 (0.704, 0.845)	0.845 (0.716, 0.998)
Concordance	0.874	0.872	0.835

Note. Models also control for birth cohort

The results from the model of non-Hispanic whites were the same, likely due to the overwhelming proportion of non-Hispanic whites in the analytic sample. While partnership status, employment status, wealth, being a Medicare recipient, BMI, having high blood pressure, and having arthritis registered significance in the model for non-Hispanic blacks as well, there were some discrepancies in other variables. Income, alcohol consumption, and having a cardiovascular disease were no longer significant at the five-percent level. Former smokers (in addition to current smokers) were found to be at an increased risk of diabetes onset compared to never smokers among non-Hispanic blacks. Additionally, regular exercise was associated with a decreased risk of diabetes onset among non-Hispanic blacks.

We re-estimated the three models in Table 2, but included the diabetes PGS and ancestry-specific principal components. The results from these runs are in Table 3. For the analytic sample, the estimated hazard ratio (HR) of the diabetes PGS was 1.16 (95% confidence interval [CI] = 1.12, 1.20), suggesting that a one SD increase in the diabetes PGS increased the risk of diabetes by 16% while holding adjusted covariates constant. In these analyses, the diabetes PGS was statistically significant for both non-Hispanic whites (HR = 1.38, 95% CI = 1.30, 1.46) and non-Hispanic blacks (HR = 1.22, 95% CI = 1.06, 1.40). The HR for the analytic sample did not fall between those obtained from the stratified analyses for non-Hispanic whites and non-Hispanic blacks. The stratified models implicitly allowed for interactions between race and all other covariates in the model. Thus, it is possible that allowing for these interactions affected the coefficient estimates for the diabetes PGS.

**Table 3:** Hazard Ratios from Multivariate Cox Regressions Model With the Diabetes PGS Included as a Covariate

Characteristic	Hazard ratio (95% confidence interval)		
	Analytic sample (*n* = 15,190)	Non-Hispanic whites (*n* = 12,090)	Non-Hispanic blacks (*n* = 3100)
Diabetes PGS	1.159 (1.116, 1.204)	1.380 (1.300, 1.464)	1.219 (1.059, 1.402)
Male	1.075 (0.986, 1.172)	1.096 (0.992, 1.211)	0.964 (0.802, 1.158)
Non-Hispanic white	0.735 (0.659, 0.820)	NA	NA
Foreign-born	0.912 (0.749, 1.110)	0.905 (0.713, 1.148)	1.076 (0.761, 1.523)
Education			
Less than high school (reference group)	1	1	1
High school/GED	1.032 (0.916, 1.162)	1.006 (0.873, 1.159)	1.002 (0.803, 1.251)
Some college	1.014 (0.890, 1.156)	1.012 (0.867, 1.181)	0.976 (0.759, 1.255)
College or above	0.883 (0.764, 1.021)	0.883 (0.745, 1.047)	0.911 (0.679, 1.224)
Married/partnered	1.607 (1.456, 1.774)	1.594 (1.418, 1.793)	1.536 (1.283, 1.838)
Employment status			
Employed (reference group)	1	1	1
Unemployed	0.979 (0.771, 1.242)	0.818 (0.597, 1.121)	1.339 (0.924, 1.942)
Retired	0.602 (0.542, 0.668)	0.571 (0.505, 0.645)	0.726 (0.594, 0.887)
Disabled	1.965 (1.527, 2.527)	1.829 (1.285, 2.604)	2.227 (1.518, 3.267)
Not in labor force	0.931 (0.781, 1.110)	0.873 (0.716, 1.065)	1.184 (0.788, 1.778)
Income	0.904 (0.863, 0.947)	0.891 (0.842, 0.943)	0.946 (0.871, 1.026)
Wealth	0.922 (0.901, 0.943)	0.913 (0.887, 0.940)	0.949 (0.912, 0.986)
Medicare	0.190 (0.168, 0.214)	0.176 (0.153, 0.204)	0.251 (0.198, 0.318)
Medicaid	1.019 (0.853, 1.218)	1.005 (0.774, 1.306)	1.130 (0.889, 1.435)
Health insurance	1.032 (0.948, 1.123)	0.991 (0.900, 1.092)	1.181 (0.990, 1.409)
BMI	1.086 (1.079, 1.093)	1.098 (1.090, 1.107)	1.056 (1.044, 1.068)
Regular exerciser	1.000 (0.921, 1.087)	1.079 (0.982, 1.185)	0.833 (0.698, 0.994)
Smoking status			
Never smoker (reference group)	1	1	1
Current smoker	1.370 (1.211, 1.550)	1.380 (1.193, 1.597)	1.330 (1.050, 1.683)
Former smoker	1.070 (0.983, 1.165)	1.021 (0.926, 1.125)	1.239 (1.036, 1.483)
Heavy drinker	0.824 (0.714, 0.952)	0.797 (0.674, 0.942)	0.943 (0.706, 1.261)
High blood pressure	1.851 (1.699, 2.017)	1.871 (1.699, 2.060)	1.582 (1.304, 1.920)
Cardiovascular disease	1.116 (1.019, 1.221)	1.157 (1.045, 1.281)	1.054 (0.857, 1.295)
Arthritis	0.796 (0.735, 0.862)	0.774 (0.706, 0.849)	0.853 (0.721, 1.008)
Concordance	0.877	0.876	0.838

Note. Models also control for birth cohort and ancestry-specific principal components

As before, the variables that were significant in the overall model were also significant in the model for non-Hispanic whites, but these did not necessarily line up with the variables that were significant in the model for non-Hispanic blacks. Again, income, alcohol consumption, and having a cardiovascular disease were not significant for diabetes onset among non-Hispanic blacks, while being a former smoker (compared to being a never smoker) and being a regular exerciser were significant. Additionally, arthritis was no longer a significant health comorbidity for non-Hispanic blacks once the diabetes PGS was included.

The variables that were significant before, for the most part, remained significant in the corresponding models that included genetic information, so the inclusion of the diabetes PGS and ancestry-specific principal components generally did not change the significance of any of the associations between the other characteristics and diabetes onset. However, these models informed us that the PGS was also a significant variable for diabetes onset and that its relationship should not be ignored.

Furthermore, models with the diabetes PGS and ancestry-specific principal components performed better than those without them. Concordance was consistently higher for the models in Table 3 than the corresponding models in Table 2. Analyses of deviance were computed for corresponding models in Tables 2 and 3, and these results are presented in Table 4. For the analytic sample, the non-Hispanic white subset, and the non-Hispanic black subset, the tests were statistically significant. That is, the inclusion of these genetic components significantly improved model fit in the explanation of diabetes onset.

**Table 4:** Test Statistics from Analyses of Deviance Comparing Models Without and With the Diabetes PGS Included as a Covariate

	Analytic sample (*n* = 15,190)	Non-Hispanic whites (*n* = 12,090)	Non-Hispanic blacks (*n* = 3100)
Without diabetes PGS	Log lik = −33,196	Log lik = −24,940	Log lik = − 6368
With diabetes PGS	Log lik = − 33,159	Log lik = − 24,865	Log lik = − 6364
Test statistic	χ2 = 74.41	χ2 = 149.41	χ2 = 9.09

Note. Statistically significant differences between models without and with the diabetes PGS for each sample at the *p* = 0.05 level

## Discussion

In the current study, we utilized a national population-based sample of older Americans to explore diabetes onset and better understand the effects of genetic endowment and time-varying behavioral characteristics commonly associated with diabetes. Models with the genetic information performed significantly better than models without it. The diabetes PGS was consistently statistically significant with diabetes onset after testing different operationalizations and adjusting for a range of characteristics. Respondents with a higher genetic propensity for diabetes were at higher risk of diabetes, irrespective of the other characteristics we included in our model. We found a number of these other characteristics to be statistically significantly associated with diabetes onset, including sociodemographics, economic well-being, behavior and lifestyle, and health comorbidities. Similar behavioral variables were also found to be significant in other population-based studies [27].

Our cumulative hazard curves demonstrated that diabetes onset differed between non-Hispanic whites and non-Hispanic blacks, both in rate of onset as well as overall levels of onset, which has been observed previously in the literature [28, 29]. In stratified models by race, we found that the association between the diabetes PGS and onset of diabetes to be statistically significant among both non-Hispanic white and non-Hispanic black respondents, but the relationship was stronger among non-Hispanic whites. Although PGSs were calculated separately for European and African ancestry groups, the GWAS meta-analysis used to estimate SNP weights were derived from analyses based on European ancestry groups; thus, the predictive power of the PGSs for African ancestry groups may vary [13, 17]. Therefore, the weaker relationship of the diabetes PGS among the non-Hispanic black sample could be due to a myriad of factors. For example, it could be an artifact of how the PGS was calculated, it could be due to the smaller sample size for non-Hispanic black respondents, or possibly a true weaker association between the diabetes PGS and diabetes onset. Extending GWASs to other ancestry groups is essential for a better understanding of how well these PGSs can actually perform for groups that are not non-Hispanic white.

Regardless of race, genetics were associated with diabetes onset. However, this should not downplay the role of behavioral or lifestyle characteristics. These behavioral and lifestyle characteristics “ultimately interact with risk alleles in susceptibility genes to initiate common forms of [diabetes]” [30]. For both non-Hispanic whites and non-Hispanic blacks, BMI was significantly associated with a higher propensity of diabetes onset, as was being a current smoker (compared to being a never smoker). Interestingly, being a former smoker (compared to being a never smoker) was also associated with a higher propensity of diabetes onset for non-Hispanic blacks. Heavy drinking was associated with a decreased risk of diabetes onset for non-Hispanic whites but not non-Hispanic blacks, while exercising was associated with a decreased risk of diabetes onset for non-Hispanic blacks but not non-Hispanic whites. These results demonstrate the potential ability of behavioral characteristics as a mechanism for delaying or preventing diabetes onset, and lifestyle interventions have indeed been useful in prevention of type II diabetes [31, 32]. However, differences between non-Hispanic whites and non-Hispanic blacks in stratified models reveal the potential need for targeted interventions, as well as the need to expand this line of research to other race and ethnic population segments.

There are a few limitations to note. In our study, the analytic sample comprised of respondents who consented and provided DNA samples for genotyping. The Additional file 1 presents the summary characteristics of our analytic sample and the summary characteristics of the complete HRS sample. Our analytic sample differed significantly when compared to the complete HRS sample, which perhaps is not surprising, as there was selection into the genetic sample. For example, respondents in the analytic sample were more likely to be younger (56.53 years vs. 62.76 years) and have higher BMI (27.82 kg/m^2^ vs. 27.05 kg/m^2^). This is a caveat that has been noted in several prior studies [33–35], and unfortunately cannot be rectified with the use of weights. These differences should be taken into account when considering the results and interpretations of our findings.

Mortality selection could also be a concern, as respondents had to survive to age 50 (or be the spouse of someone who survived to age 50) in order to be in the HRS sampling frame. While this is an issue with all studies using the HRS, respondents in studies also using the genetic component had to survive to 2006-2012 in order to be included for potential genetic sampling.

Another caveat is that the survey question used in the HRS to assess regular physical activity changed after wave 6. In our analysis, a respondent was considered a regular exerciser during waves 1-6 if they reported vigorous physical activity 3+ times per week or, for waves 7-12, if they reported vigorous physical activity at least once per week. We opted to classify vigorous physical activity based on how it was defined in the wave by HRS.

## Conclusion

Despite the limitations, this paper has shown the importance of looking at the effects of genetic and behavioral characteristics together, and that both are necessary in understanding the etiology of diabetes. Although previous papers have examined them together, the advantage of this paper is that we studied their relationship in both non-Hispanic white and non-Hispanic black respondents using a national population-based study. Our findings suggest that although genetic variants are associated with diabetes onset, behavioral and lifestyle characteristics remain an important part of diabetes management. BMI, smoking, alcohol, and exercise were all found to be significant in various specifications of our models. Thus, despite the statistically significant role genetic endowment plays in diabetes onset, individuals might still be able to reduce their risk by engaging in protective behaviors, which has substantial clinical relevance.

Diabetes is a multifaceted trait that has both a heritable and lifestyle component. A 2015 review by Prasad and Groop [36] reported that the heritability of type two diabetes mellitus varied between 25 and 80%, depending on the length of follow-up, which may indicate a change in heritability with age and thus the changing importance of modifiable risk factors for diabetes onset. In the context of our findings that both lifestyle factors and genetic risk play a role in diabetes onset, it is important to target lifestyle factors that may mitigate the role of genetic endowment. Thus, future studies should examine gene-environment interactions in the onset of diabetes. Understanding the contribution of lifestyle factors over the lifespan to epigenetic changes in the expression of genetic risk for diabetes would be a valuable contribution to this line of work.

## Supplementary information

**Additional file 1.** Summary Characteristics for the Analytic and Complete Samples. This additional file demonstrates how the analytic sample used for this study differed from the complete HRS sample.

## Data Availability

The datasets used for the current study are publicly available in the National Archive of Computerized Data on Aging repository: https://hrs.isr.umich.edu/data-products.

## Abbreviations

BMI: Body mass index
CI: Confidence interval
DNA: Deoxyribonucleic acid
GWAS: Genome-wide association study
HR: Hazard ratio
HRS: Health and retirement study
NIA: National Institute on Aging
PGS: Polygenic score
SD: Standard deviation
SNP: Single nucleotide polymorphism
US: United States

## Footnotes

1 Household income was the sum of all income in a household, including respondent’s and spouse’s income from wages, pension and annuity, social security, disability, and retirement, unemployment and workers compensation, other government income, as well as household capital and other income.

2 Household wealth was the net value of total household wealth including primary residence, other real estate, transportation, businesses, stocks and bonds, checking and savings accounts, bonds, total mortgage, other home loans, debt, and individual retirement accounts.
