# Supplementary material for: Association of genetic and behavioral characteristics with the onset of diabetes
Source: BMC Public Health. 2019 Oct 15;19:1297. doi: 10.1186/s12889-019-7618-z (PMC6794810; doi:10.1186/s12889-019-7618-z)
Supplement: Supplementary file 1 — Additional file 1. Summary Characteristics for the Analytic and Complete Samples. This additional file demonstrates how the analytic sample used for this study differed from the complete HRS sample. [file 12889_2019_7618_MOESM1_ESM.docx]

Summary Characteristics for the Analytic and Complete Samples.

| Characteristic | Mean or % | |
| --- | --- | --- |
|  | Analytic sample  (n = 15,190) | Complete HRS sample  (n = 37,495) |
| Age, mean | 56.53 | 60.24 |
| Male, % | 42.04 | 43.82 |
| Non-Hispanic white, % | 79.59 | 68.20 |
| BMI, mean | 27.82 | 27.36 |
| Regular exerciser, % | 32.12 | 28.83 |
| Smoking status, % |  |  |
| Current smoker | 21.59 | 21.73 |
| Former smoker | 36.08 | 36.47 |
| Never smoker | 42.21 | 41.63 |
| Heavy drinker, % | 9.33 | 8.39 |

**Note**. Statistically significant differences between the analytic sample and excluded respondents were observed for all characteristics at the p = 0.05 level.
